# Falsely elevated serum estradiol due to heterophile antibody interference: a case report

**DOI:** 10.20945/2359-3997000000324

**Published:** 2021-02-15

**Authors:** Paul Atkins, Andre Mattman, David Thompson

**Affiliations:** 1 University of British Columbia Department of Medicine Division of Endocrinology Vancouver Canada Division of Endocrinology, Department of Medicine, University of British Columbia, Vancouver, British Columbia, Canada.; 2 University of British Columbia Department of Pathology and Laboratory Medicine Vancouver Canada Department of Pathology and Laboratory Medicine, University of British Columbia, Vancouver, British Columbia, Canada.

## Abstract

Falsely elevated estradiol is rare, may result from heterophile antibody interference, and can result in unnecessary investigation and intervention. We present the case of a 56-year-old female with falsely elevated estradiol levels inconsistent with her overall clinical picture, which ultimately led to an unnecessary surgical procedure. With the use of alternative analytical platforms and a heterophile antibody blocking agent, we determined the false elevation was due to heterophile antibody interference. Clinicians must suspect and investigate for laboratory error when the clinical picture contradicts laboratory results.

## INTRODUCTION

The optimal management of patients in endocrinology often relies upon the accuracy and validity of biochemical assays. When results are discordant with the clinical picture, laboratory error must be suspected by the clinician. While pre-analytical errors are most commonly encountered, analytical errors related to interfering endogenous or exogenous substances are a significant cause of morbidity related to unnecessary investigation, intervention, and patient anxiety (1,2). As simple, cheap, high-throughput tests, immunoassays have become increasingly common and often guide clinical decisions in endocrinology (1). Two common formats are employed: immunometric and competitive. Immunometric assays measure an analyte by way of forming a ‘sandwich’ between an immobilized solid phase antibody and a measurable labelled antibody, each binding to separate epitopes on the analyte of interest (1,3). For analytes such as estradiol that are too small to practically allow for two separate antibody binding sites, competitive immunoassays are utilized. In this format, a known quantity of labeled analyte competes with patient analyte for binding to a solid phase antibody, and the concentration of patient analyte can be extrapolated from the measurement of labeled analyte (1). In contrast to immunometric assays in which the label signal is proportional to the analyte concentration, the label signal of a competitive assay is inversely proportional to the analyte concentration (1,2).

Despite significant advances in the reliability of immunoassays, analytical interference remains a major and underestimated problem. Prevalence ranges from 0.05% to 6%, depending on the specific assay (1). Interfering substances can impair the interaction between test antibody and analyte. Examples of such substances include heterophile antibodies, therapeutic antibodies, autoantibodies, rheumatoid factor, or cross-reacting substances that compete with the analyte for antibody binding (4,5). Interfering substances may also impact other components of the assay; biotin supplementation is a frequent cause of interference in streptavidin-biotin based immunoassays (6). Cross-reacting substances represent the most common analytical interference affecting competitive immunoassays, though heterophile antibodies and other causes of interference are possible (2,7).

False elevation of estradiol due to analytical interference is rare and is most commonly associated with cross-reacting substances such as the selective estrogen receptor degrader fulvestrant (8) or the aromatase inhibitor exemestane (9). To the best of our knowledge, only five cases of falsely elevated estradiol definitively due to a heterophile antibody have been reported previously (10–13). Here, we report a case of falsely elevated estradiol due to heterophile antibody interference that led to considerable morbidity and an unnecessary surgical procedure.

## CASE

A 56-year-old female was referred in the fall of 2019 for assessment of persistent severely elevated estradiol levels, despite having undergone a recent bilateral oophorectomy. Her past medical history included a history of hysterectomy performed at age 40 for bulky leiomyomas, prior Grave's disease treated remotely with radioactive iodine, and mitral valve prolapse. Her medications at the time of referral included synthroid, liothyronine, propanolol, citalopram, trazodone, and topical conjugated equine estrogen. Family history was non-contributory.

In 2018, the patient was seen by a naturopath who performed dried urine mass spectrometry testing (14) as part of a workup for low libido and fatigue. An elevated urinary estradiol of 8.56 ng/mg (luteal: 1.8-4.5; postmenopausal: 0.2-0.7) was discovered; however, the test is intended to be taken on days 19-22 of the menstrual cycle (14) and due to the patient's prior hysterectomy no menses were present to allow identification of the luteal phase. This raises the possibility that the sample was obtained during the ovulatory peak. She reported taking no hormonal medications or supplements at this time.

Further workup at a local laboratory yielded the following results: estradiol 2337 pmol/L (mid-follicular 110-184; ovulatory peak 550-1650; mid-luteal 550-845; postmenopausal ≤ 220), LH 10.4 IU/L (follicular/luteal <13.0; mid-cycle 14.0-100.0; postmenopausal 15.0-65.0), FSH 16.3 IU/L (follicular/luteal <9.0; mid-cycle 4.0-20.0; postmenopausal 20.0-135.0), prolactin 9.9 ug/L (female 2.7-26.0; postmenopausal 1.8-17.9), and CA-125 6 kU/L (<36). Estradiol was repeated on several occasions in the remainder of 2018, measuring as 1312 pmol/L and later 4273 pmol/L. In February 2019, estradiol was found to be severely elevated to 8069 pmol/L. At that time, FSH was 8.6 IU/L, progesterone 32.5 nmol/L (follicular <5.0; luteal 16-95; postmenopausal <5.0), and carcinoembryonic antigen (CEA) 2 ug/L (0–5). In April 2019, estradiol remained high at 3214 pmol/L, with LH 9.1 IU/L, FSH 9.6 IU/L, progesterone <0.7, and testosterone 0.6 nmol/L (female premenopausal 0.4-2.1; postmenopausal <0.2-1.7).

At this stage, a pelvic ultrasound was performed and revealed a right ovarian cyst measuring 3.4cm in diameter. On the basis of severely elevated estradiol and the cystic lesion, a granulosa cell tumor of the ovary was suspected and the patient underwent a bilateral salpingo-oophorectomy in May 2019; however, the pathology returned as benign. Post-operatively, the patient noted new onset hot flashes and vaginal atrophy that was relieved by topical estrogen, clinically compatible with a transition from pre- or perimenopause to surgical menopause. Investigations in July 2019 revealed the following: estradiol 1353 pmol/L, LH 26.3 IU/L, FSH 64.1 IU/L, testosterone 0.5 IU/L, and progesterone <0.7 nmol/L. Her estradiol was repeated numerous times over the subsequent 6 months leading up to the current referral, measuring between 1055-1607 pmol/L. All estradiol measurements thus far had been obtained from the same laboratory, using the same methodology (Siemens ADVIA CENTAUR^®^). Despite a bilateral oophorectomy, clinical evidence of hypoestrogenism, and appropriate rise in FSH consistent with surgical menopause, estradiol levels remained significantly elevated post-operatively. On this basis, a laboratory error was suspected.

A sample was then sent to another laboratory using a distinct immunoassay method (Roche Diagnostics, electrochemiluminescence, Cobas^®^ e601) and an estradiol level of 27 pmol/L was measured. Subsequently, in the original laboratory utilizing the Siemens ADVIA CENTAUR^®^ method, pre-incubation of the plasma sample with a Scantibodies^®^ heterophilic blocking tube (HBT) resulted in a significant (80.4%) reduction in measured estradiol concentration from 1207 pmol/L pre-HBT to 236 pmol/L post-HBT. In other laboratories, using the Abbott^®^ Alinity and Ortho Clinical Diagnostics^®^ Vitros5600 methods, estradiol was measured as below the limit of quantification (<40 pmol/L) and 60 pmol/L, respectively, without HBT pre-incubation. Based upon these clinically concordant low normal values found on alternative methods and the significant reduction post-HBT, we concluded that the original estradiol measurements had been artefactually high due to interference by heterophile antibodies. Notably, a review of previous investigations revealed normal results for rheumatoid factor, immunoglobulin levels, and serum protein electrophoresis. On further history, the patient denied any significant contact with animals or prior administration of therapeutic immunoglobulins.

## DISCUSSION

We present the case of a 56-year-old female with high estradiol levels inconsistent with the clinical picture, raising a suspicion of laboratory error. In the pre-surgical period, severely elevated serum estradiol measurements were incongruent with associated unsuppressed gonadotropin values. Following bilateral oophorectomy, estradiol values remained severely elevated and in disagreement with both clinical evidence of hypoestrogenism and the rise in FSH consistent with surgical menopause. Following the use of alternative analytical platforms, and the observation of a marked reduction in estradiol measurement on the original method using samples pre-treated with a heterophile-blocking agent, we determined that the original elevations in estradiol were spurious results attributable to heterophile antibody interference.

Heterophile antibodies are defined as endogenous antibodies that bind external antigens (15). When they bind reagent immunoglobulins and other components used in immunoassays, they can be a source of interference indiscriminately affecting one or more methods. As these immunoassays are used to measure a variety of analytes, such as hormones, tumour markers, markers of cardiac injury, or therapeutic drugs, all are subject to spurious interference (15). Such antibodies may occur naturally without an identifiable cause, or may result from infection, vaccination, contact with animals, or the use of animal immunoglobulins therapeutically or for in vivo diagnostic testing (eg. immunoscintigraphy) (15,16). Autoantibodies such as rheumatoid factor may also act as interfering heterophilic antibodies (15). Classically, heterophile antibodies are polyspecific and bind with weak affinity to numerous antigens. Also described are human anti-animal antibodies (HAAA) that bind with high specificity and affinity to an animal antigen (2,15), and these are thought to more commonly result from direct exposure to animal immunoglobulin injected for therapeutic or diagnostic purposes (2). Though the prevalence of potentially interfering heterophile antibodies in the general population is estimated to be 30-40% based on serum samples (17,18), analytically important interferences are reported in only 0.5%–3% of specimens (5,19,20).

When suspected, multiple verification strategies are available to support or exclude the presence of heterophile antibodies. The investigation can be repeated on alternative analytical platforms, the analysis can be repeated following treatment with heterophile-blocking agents, polyethylene glycol may be used to precipitate potential interfering antibodies, or serial dilution can be performed to examine for non-linearity (2). We employed the first two strategies, which revealed normal results on three alternative platforms and a significant reduction in estradiol following treatment with a heterophile-blocking agent. While liquid chromatography-tandem mass spectrometry (LC-MSMS) is a highly accurate method impervious to heterophile antibody interference, it is not readily available and was considered unnecessary in this case. Table 1 provides an overview of the above verification strategies and how they can be applied to determine the presence of heterophile antibodies or other causes of clinically discordant immunoassay results.

**Table 1:** Potential causes and verification of clinically discordant immunoassay results

		True result	Macro-analyte	Hook Effect	Heterophile Ab	Biotin	Other Error (Rare)
Clinically discordant result affects							
	Only a single immunoassay test		✓	✓	✓	✓	✓
	Multiple immunoassay tests from same vendor				P	P	P
	Multiple methodologies from different vendors	✓	P			P	
Verification strategy							
	Alternative Method	S	Possible	Err Detect	Err Detect	Err Detect	Err Detect
	Heterophile Blocking Tube	S	S	S	Err Detect	S	S
	Serial Dilution	S	S	Err Detect	Possible	Err Detect	Possible
	PEG Precipitation	S	Err Detect	S	Possible	S	S
	Mass Spectrometry	S	Err Detect	Err Detect	Err Detect	Err Detect	Err Detect

Table 1 provides an overview of potential causes of clinically discordant immunoassay results and relevant verification strategies to determine the cause. The checkmarks represent likely scenarios while the P indicates possible scenarios. A true result may be explained by the presence of a rare genetic and/or pathologic syndrome. Macro-analyte refers to formation of an antibody-analyte complex, such as macroprolactin. A single nucleotide polymorphism (SNP) in the region of an analyte epitope that affects reagent antibody binding is one possible rare error. Endogenous anti-streptavidin antibodies in a test method that is dependent on streptavidin-biotin binding is another.

*Ab* Antibody; *PEG* polyethylene glycol; *S* verification step gives the same result as the original test; *Err Detect* verification step detects the error; *Possible* verification step may or may not detect the error.

Though heterophile antibodies most commonly interfere with immunometric (sandwich) immunoassays, they can be a cause of interference in competitive immunoassays (2). Because a strong interaction between analyte and test antibody exists in competitive assays, weak heterophile antibodies are only able to directly interfere at unusually high concentrations (7). However, heterophile antibodies may still indirectly interfere by binding the capture antibody, the antigen label (eg chemical, enzyme, radioactive), conjugate, or other parts of the detection system (eg streptavidin coated capture beads) all of which suppress the reaction signal (7). In a competitive immunoassay, a suppressed signal connotes a high concentration of the measured analyte. Chemiluminescent immunoassays (CLIA) and other more modern forms of competitive immunoassays are thought to be more susceptible than radioimmunoassays (RIA), due to the more extensive mixture of ingredients providing potential binding sites for interference (7). In our case, the falsely elevated estradiol was related to use of the Siemens ADVIA CENTAUR^®^ method, a competitive CLIA. Because alternative estradiol analytical platforms as well as other CLIA assays on the same platform all generated clinically concordant results, the heterophile antibody interference may have been specifically directed towards estradiol reagent immunoglobulins unique to the Siemens ADVIA CENTAUR^®^ Estradiol method. These reagent immunoglobulins are distinct as compared to those present in other Siemens ADVIA CENTAUR assays, or those in alternative estradiol methods such as the Roche method. This would account for the presence of interference selectively in the Siemens ADVIA CENTAUR^®^ method.

False elevation of estradiol due to immunoassay interference is rarely reported in the literature. It is most often related to cross-reactivity from drugs sharing a similar structure to estradiol, namely fulvestrant (8) and exemestane (9). It has also been reported in relation to biotin supplementation in the setting of a streptavidin-biotin based immunoassay (21). To the best of our knowledge, five previously reported cases were definitively related to heterophile antibodies: one case of a heterophile antibody against bovine alkaline phosphatase (ALP) affecting a competitive ALP-containing CLIA (Beckman Coulter UniCel Dxl 800) (13), one case of an IgA lambda heterophile antibody found to bind the ^125^I-labeled tracer of a competitive RIA (12), and three cases of interfering anti-rabbit IgG heterophile antibodies (10,11). We report a case of heterophile antibody interference causing false estradiol elevation in the setting of a competitive CLIA (Siemens ADVIA CENTAUR^®^)

## CONCLUSION

Falsely elevated estradiol is a rare but not insignificant phenomenon that may result from heterophile antibodies and may lead to unnecessary investigation or intervention. In the present case, a heterophile antibody caused a false elevation of estradiol inconsistent with the overall clinical picture, which led to an unnecessary surgical procedure and consequent surgical menopause. Clinicians must always exercise caution when interpreting laboratory values and appreciate the potential harm that can occur due to laboratory error. In particular, clinicians must have a high index of suspicion when the clinical picture contradicts laboratory results.

## References

[1] Sturgeon CM, Viljoen A (2011). Analytical error and interference in immunoassay: minimizing risk. Ann Clin B Biochem.

[2] Klee GG (2004). Interferences in hormone immunoassays. Clin Lab Med.

[3] Bolstad N, Warren DJ, Nustad K (2013). Heterophilic antibody interference in immunometric assays. Best Pract Res Clin Endocrinol Metab.

[4] Langlois F, Moramarco J, He G, Carr BR (2017). Falsely Elevated Steroid Hormones in a Postmenopausal Woman Due to Laboratory Interference. J Endocr Soc.

[5] Tate J, Ward G (2004). Interferences in immunoassay. Clin Biochem Rev.

[6] Piketty ML, Polak M, Flechtner I, Gonzales-Briceño L, Souberbielle JC (2017). False biochemical diagnosis of hyperthyroidism in streptavidin-biotin-based immunoassays: the problem of biotin intake and related interferences. Clin Chem Lab Med.

[7] Levinson SS, Miller JJ (2002). Towards a better understanding of heterophile (and the like) antibody interference with modern immunoassays. Clin Chim Acta.

[8] Owen LJ, Monaghan PJ, Armstrong A, Keevil BG, Higham C, Salih Z (2019). Oestradiol measurement during fulvestrant treatment for breast cancer. Br J Cancer.

[9] Mandic S, Kratzsch J, Mandic D, Debeljak Z, Lukic I, Horvat V (2017). Falsely elevated serum oestradiol due to exemestane therapy. Ann Clin Biochem.

[10] Check JH, Ubelacker L, Lauer CC (1995). Falsely elevated steroidal assay levels related to heterophile antibodies against various animal species. Gynecol Obstet Invest.

[11] Kairemo KJ, Kahn JA, Taipale PJ (1999). Monoclonal gammopathy may disturb oestradiol measurement in the treatment and monitoring of in-vitro fertilization: case report. Hum Reprod.

[12] Gordon DL, Holmes E, Kovacs EJ, Brooks MH (1999). A spurious markedly increased serum estradiol level due to an IgA lambda. Endocr Pract.

[13] Maharjan AS, Wyness SP, Ray JA, Willcox TL, Seiter JD, Genzen JR (2019). Detection and characterization of estradiol (E2) and unconjugated estriol (uE3) immunoassay interference due to anti-bovine alkaline phosphatase (ALP) antibodies. Pract Lab Med.

[14] Newman M, Pratt SM, Curran DA, Stanczyk FZ (2019). Evaluating urinary estrogen and progesterone metabolites using dried filter paper samples and gas chromatography with tandem mass spectrometry (GC–MS/MS). BMC Chem.

[15] Dasgupta A (2019). Biotin and Other Interferences in Immunoassays: A Concise Guide.

[16] Henry N, Sebe P, Cussenot O (2009). Inappropriate treatment of prostate cancer caused by heterophilic antibody interference. Nat Clin Pract Urol.

[17] B Boscato LM, Stuart MC (1986). Incidence and specificity of interference in two-site immunoassays. Clin Chem.

[18] Ward G, McKinnon L, Badrick T, Hickman PE (1997). Heterophilic antibodies remain a problem for the immunoassay laboratory. Am J Clin Pathol.

[19] Preissner CM, O’Kane DJ, Singh RJ, Morris JC, Grebe SK (2003). Phantoms in the assay tube: heterophile antibody interferences in serum thyroglobulin assays. J Clin Endocrinol Metab.

[20] Howanitz JH (2008). Immunoassay interference by endogenous antibodies: approved guideline.

[21] Batista MC, Ferreira CES, Faulhaber ACL, Hidal JT, Lottenberg SA, Mangueira CLP (2017). Biotin interference in immunoassays mimicking subclinical Graves’ disease and hyperestrogenism: a case series. Clin Chem Lab Med.

